# Mulberrofuran A: A Multifunctional 2-Arylbenzofuran Flavonoid—Insights into Pharmacological Actions, Molecular Mechanisms, and Therapeutic Potential

**DOI:** 10.3390/molecules31101755

**Published:** 2026-05-20

**Authors:** Fan Qiu, Cunbao Ling, Shaoyue Wang, Siyuan Chen, Liping Liu, Xuan Wang, Yuping Chen

**Affiliations:** 1 School of Traditional Chinese Medicine, Jiangsu Medical College, Yancheng 224005, China; echo_qf@163.com (F.Q.); 202442093@jsmc.edu.cn (S.W.); 202442103@jsmc.edu.cn (S.C.); liuliping1487@163.com (L.L.); 2 School of Basic Medical Science, Jiangsu Medical College, Yancheng 224005, China; eesms1000@163.com; 3 Science and Technology Division, Jiangsu Medical College, Yancheng 224005, China

**Keywords:** Mulberrofuran A, *Morus alba*, type 2 diabetes, anti-inflammatory, natural products, arachidonic acid metabolism

## Abstract

Mulberrofuran A (MFA), a natural product originally isolated from the root bark of *Morus alba* L. (Sang-Bai-Pi), is a structurally distinctive mulberry-derived 2-arylbenzofuran bearing a prenyl-related side chain. Although MFA has attracted attention because of its phytochemical uniqueness and reported biological relevance, the available evidence specific to MFA remains limited and fragmented. In addition, pharmacological interpretations are often complicated by the frequent use of indirect evidence derived from structurally related mulberrofuran analogues, other arylbenzofurans, or complex Morus extracts. This review critically summarizes current knowledge on the chemistry, occurrence, and biological relevance of MFA, while explicitly distinguishing direct MFA-specific evidence from indirect and contextual evidence. Available studies suggest that MFA may be associated with antimicrobial activity and modulation of arachidonic acid-related inflammatory pathways, whereas its putative roles in metabolic regulation, cardiovascular protection, antiviral activity, antioxidant effects, and anticancer relevance are currently supported mainly by structurally related compounds or broader mulberry literature rather than robust MFA-specific validation. We further discuss the limitations of the current evidence base, including methodological heterogeneity, incomplete statistical reporting, the lack of pharmacokinetic and toxicity data, and the absence of clinical validation. Rather than establishing MFA as a confirmed therapeutic agent, the available literature supports its consideration as an emerging natural product candidate that warrants rigorous chemical, pharmacological, and translational investigation.

## 1. Introduction

*Morus alba* L. (white mulberry) has long been used in traditional Chinese, Korean, and Japanese medicine for the management of disorders such as inflammation, diabetes, hypertension, and liver dysfunction [1]. Different medicinal parts of the plant, including the root bark, leaves, fruits, and twigs, have been documented in traditional pharmacopoeias and investigated for a broad range of biological effects [2,3]. Phytochemical studies have revealed that Morus species contain structurally diverse constituents, including flavonoids, stilbenoids, prenylated phenolics, Diels–Alder-type adducts, and arylbenzofuran derivatives, many of which are considered important contributors to the medicinal value of the plant [1,2,4,5].

Among these constituents, 2-arylbenzofurans represent a distinctive class of mulberry-derived natural products, particularly enriched in the root bark of *M. alba* [2,6,7]. Mulberrofuran A (MFA), first reported in 1978 [6], is one of the characteristic members of this class and has attracted attention because of its unusual structural features and potential biological relevance. Early studies associated MFA with antimicrobial activity and reported effects on arachidonic acid (AA) metabolism [3]. However, these observations remain limited in number, and the depth of compound-specific pharmacological investigation is still insufficient compared with that for other Morus-derived constituents [8,9].

In recent years, increasing attention has been given to structurally related mulberrofurans, arylbenzofurans, and Morus-derived phytochemicals with reported antidiabetic [8,10,11], anti-inflammatory [12,13], antiviral [14,15], cardiovascular [16], neuroprotective [17], and anticancer-related activities [18,19]. While these studies provide useful mechanistic and structure-related context, they do not necessarily constitute direct evidence for MFA itself. In particular, biological activities observed in crude extracts, multi-component fractions, or related analogues cannot be directly attributed to MFA without compound-specific isolation, quantitative evaluation, and experimental validation. Failure to distinguish between these evidence levels may lead to overinterpretation of MFA’s pharmacological potential.

Although several reviews have summarized the phytochemistry and pharmacology of Morus species or mulberry-derived compounds more broadly [1,2,3,11], no review to date has critically focused on MFA while explicitly differentiating direct MFA-specific evidence from indirect evidence derived from related compounds and extracts. This distinction is especially important because the currently available literature on MFA is limited, heterogeneous, and uneven in methodological quality.

Therefore, the aim of the present review is not simply to catalogue reported activities, but to critically evaluate the current state of knowledge regarding MFA. Specifically, this review summarizes its chemical characteristics, botanical occurrence, and reported biological relevance; distinguishes direct evidence from indirect and contextual support; discusses limitations in pharmacological, pharmacokinetic, and toxicological data; and identifies major research gaps that must be addressed before any meaningful translational or therapeutic conclusions can be drawn.

## 2. Literature Search Strategy and Evidence Classification

A structured literature search was conducted to identify publications relevant to Mulberrofuran A and related evidence. Searches were performed in PubMed, Web of Science, Scopus, Google Scholar, and other accessible databases, using combinations of the terms “Mulberrofuran A”, “*Morus* furan A”, “*Morus alba*”, “mulberry”, “arylbenzofuran”, “mulberrofuran”, “antimicrobial”, “anti-inflammatory”, “antiviral”, “diabetes”, “cardiovascular”, “anticancer”, “pharmacokinetics”, and “toxicity”. Reference lists of relevant articles were also screened to identify additional publications. Although this article is a narrative review rather than a formal systematic review, the search strategy and evidence classification were designed to improve transparency and reproducibility [17,20].

Studies were considered eligible if they reported: (i) the isolation, identification, or structural characterization of MFA; (ii) direct biological or pharmacological evaluation of MFA; (iii) mechanistic information relevant to MFA; or (iv) indirect evidence from structurally related mulberrofuran analogues, arylbenzofurans, or Morus extracts that could provide contextual or hypothesis-generating support. Publications unrelated to MFA or its relevant structural/phytochemical context, duplicate reports, and studies lacking sufficient methodological detail were excluded where possible.

To improve interpretability, the included literature was classified into three evidence levels: direct MFA-specific evidence, indirect evidence from structurally related compounds, and contextual evidence from Morus extracts or broader benzofuran literature. Greater interpretive weight was assigned to direct evidence, whereas indirect and contextual evidence were used cautiously to inform mechanistic discussion and future research directions rather than to establish confirmed MFA-specific pharmacological effects. This evidence-stratified approach also aligns with evidence-mapping principles that help distinguish areas of direct support from those dominated by indirect or hypothesis-generating data [21]. To further enhance transparency, the methodological quality of key studies was systematically evaluated with respect to study design, statistical reporting, sample size, reproducibility, and risk of bias (Table S1).

## 3. Chemical Identity, Structural Context, and Isolation of Mulberrofuran A

Chemical characterization is the foundation for evaluating the biological relevance of Mulberrofuran A (MFA). MFA was originally reported as an isoprenoid-substituted 2-arylbenzofuran from the root bark of *Morus alba* L. [6]. Its chemical identity, structural relationship with other mulberrofuran analogues, and practical isolation methods are important for interpreting the available bioactivity data [6,14]. However, it should be emphasized that structural features alone cannot establish pharmacological activity. Therefore, in this section, the chemical and structural characteristics of MFA are discussed as a basis for hypothesis generation and future experimental validation rather than as direct evidence of biological efficacy. Table 1 summarizes the representative compounds within the mulberrofuran and stilbene series found in *Morus alba*.

### 3.1. Chemical Identity and Structural Context

Mulberrofuran A is chemically described as an isoprenoid-substituted 2-arylbenzofuran derivative isolated from *Morus alba* root bark [6,7]. Its molecular formula is C_25_H_28_O_4_, with a molecular weight of 392.49 g/mol. Structurally, MFA contains a relatively rigid 2-arylbenzofuran polyphenolic core and a flexible lipophilic geranyl-related side chain. The phenolic hydroxyl groups may contribute to hydrogen-bonding interactions and redox-related properties, whereas the isoprenoid side chain may increase hydrophobicity and affect interactions with lipid membranes or hydrophobic protein pockets [8,9]. A structured evidence map of the studies discussed in this review is provided in Table 2.

These structural characteristics provide a plausible chemical basis for investigating MFA in membrane-associated or enzyme-targeted biological systems. Nevertheless, such structure-based considerations should be interpreted cautiously. Increased lipophilicity or the presence of phenolic groups does not by itself confirm antimicrobial, antioxidant, or enzyme-inhibitory activity. Direct bioassays using purified MFA, appropriate controls, and quantitative potency parameters are required to determine whether these structural features translate into meaningful biological effects.

MFA belongs to a broader group of Morus-derived phenolic compounds that includes stilbenoids, moracins, mulberrofurans, prenylated phenolics, and Diels–Alder-type adducts [1,2,5,7]. Within this phytochemical network, non-prenylated 2-arylbenzofuran scaffolds such as moracin-type compounds are generally considered structurally related to more highly substituted mulberrofurans. Prenylation or geranylation of the benzofuran core contributes to structural diversity and may influence physicochemical behavior, including lipophilicity, steric profile, and potential target interactions [6,8]. As summarized in Table 2, direct MFA-specific evidence remains limited.

Other members of the mulberrofuran family, including Mulberrofuran B, G, K, and related analogues, differ in the number, position, and type of isoprenoid substituents or in the formation of more complex polycyclic architectures [7,9,12,14]. For example, Mulberrofuran G and certain kuwanon-type compounds are often described as Diels–Alder-type adducts with increased structural complexity and multiple stereochemical elements [23]. These analogues are useful for comparative discussion, but their pharmacological effects should not be assumed to be identical to those of MFA. Structural similarity can guide hypothesis generation, but direct compound-specific testing remains necessary. Most pharmacological implications currently arise from indirect evidence or contextual evidence rather than direct MFA testing (Table 2).

### 3.2. Structural Elucidation and Isolation

The structural elucidation of MFA and related mulberrofuran derivatives generally relies on a combination of high-resolution mass spectrometry (HR-MS), one-dimensional and two-dimensional nuclear magnetic resonance spectroscopy (1D/2D-NMR), and, where necessary, chromatographic comparison with reference compounds [7,23,31]. For monomeric 2-arylbenzofurans, diagnostic NMR features typically include signals corresponding to the benzofuran proton and characteristic resonances of prenyl or geranyl substituents. Long-range correlations observed in heteronuclear multiple bond correlation (HMBC) spectra are particularly useful for determining the attachment positions of isoprenoid side chains, whereas nuclear overhauser effect spectroscopy (NOESY) data may assist in assigning spatial relationships.

For more complex Diels–Alder-type adducts, structural confirmation usually requires additional attention to stereochemical assignment and ring-system connectivity [23]. Because many Morus-derived phenolics share similar chromophores and fragmentation patterns, rigorous structural identification is essential for avoiding misattribution of biological activity [1,31]. Future studies evaluating MFA should clearly report compound purity, spectral data, chromatographic conditions, and, ideally, comparison with authentic standards.

MFA has been isolated primarily from the root bark of *Morus alba* [6,7], although the distribution and quantitative abundance of MFA across different Morus species, plant parts, harvest seasons, and extraction conditions remain insufficiently characterized [1,32]. In general, Morus root bark is extracted with aqueous alcohols such as methanol or ethanol, followed by solvent partitioning and chromatographic separation [15,32]. Because MFA is an isoprenylated phenolic compound with intermediate polarity, it is expected to be enriched mainly in moderately polar organic fractions, such as ethyl acetate-soluble fractions, rather than in highly polar aqueous fractions.

Purification of MFA and related arylbenzofurans typically involves multistep chromatographic procedures, including silica gel chromatography, Sephadex LH-20, reversed-phase column chromatography, and semi-preparative HPLC [7,15,31]. Detection is commonly performed in the UV range corresponding to the conjugated arylbenzofuran chromophore, and final structural confirmation relies on MS and NMR analyses. However, published studies vary substantially in reporting extraction yield, compound purity, chromatographic conditions, and batch-to-batch consistency.

This lack of standardization has practical implications for pharmacological interpretation. When biological activity is evaluated using crude extracts or incompletely characterized fractions, it is difficult to determine whether the observed effect is attributable to MFA, other co-occurring Morus phenolics, or synergistic interactions among multiple constituents [1,9]. Therefore, future bioactivity studies should use purified MFA with clearly reported purity, stability, and quantitative concentration.

### 3.3. Chemical Knowledge Gaps

Despite the early discovery of MFA [6], several chemical questions remain unresolved. First, quantitative data on the natural abundance of MFA in different Morus species and plant parts are limited [1,32]. Second, systematic comparisons of extraction efficiency, seasonal variation, and processing effects are scarce [32]. Third, the chemical stability of purified MFA under storage, assay, and gastrointestinal conditions has not been adequately evaluated. Finally, structure–activity relationships within the mulberrofuran family remain incompletely defined [8,11], making it difficult to predict which structural elements are most important for biological activity.

Addressing these gaps is essential for improving reproducibility. Without standardized isolation, purity assessment, and quantitative reporting, pharmacological studies may be difficult to compare across laboratories. These chemical limitations also reinforce the need to distinguish direct MFA-specific evidence from data obtained using related compounds or complex Morus preparations [1,8].

## 4. Arachidonic Acid Metabolism and Inflammatory Pathways: Direct Observations and Mechanistic Hypotheses

One of the few reported direct biological observations for Mulberrofuran A (MFA) concerns its effects on arachidonic acid (AA) metabolism in platelet-based experimental systems [3,6]. Early studies suggested that MFA may influence the balance between cyclooxygenase (COX)- and lipoxygenase (LOX)-related AA metabolites [3]. However, the available evidence remains limited, and the proposed mechanism has not yet been comprehensively validated across independent experimental models [1,8]. Therefore, AA-related modulation should be interpreted as an important but preliminary mechanistic observation rather than as an established pharmacological signature of MFA.

### 4.1. Reported Effects on COX- and LOX-Related Metabolites

In rat platelet-based assays, MFA was reported to reduce the formation of 12-hydroxy-5,8,10-heptadecatrienoic acid (HHT) and thromboxane B2 (TXB2), both of which are associated with the COX branch of AA metabolism. In the same experimental context, MFA was also reported to increase the formation of 12-hydroxyeicosatetraenoic acid (12-HETE), a metabolite linked to the 12-lipoxygenase pathway [3]. These observations suggest that MFA may differentially affect AA metabolic flux rather than simply suppressing all AA-derived metabolites.

The reduction in TXB2 is pharmacologically notable because TXB2 reflects thromboxane A2 formation, which is related to platelet activation and vascular tone. Nevertheless, platelet metabolite changes alone are insufficient to establish antithrombotic or cardioprotective efficacy [26,33]. Similarly, the reported increase in 12-HETE complicates interpretation, because 12-HETE has been implicated in chemotaxis, vascular inflammation, and other context-dependent signaling processes [25,33]. Therefore, the AA-related profile of MFA should be regarded as mechanistically interesting but not yet therapeutically defined [1,3].

### 4.2. Substrate Shunting as a Proposed Mechanistic Explanation

**Figure 1:**
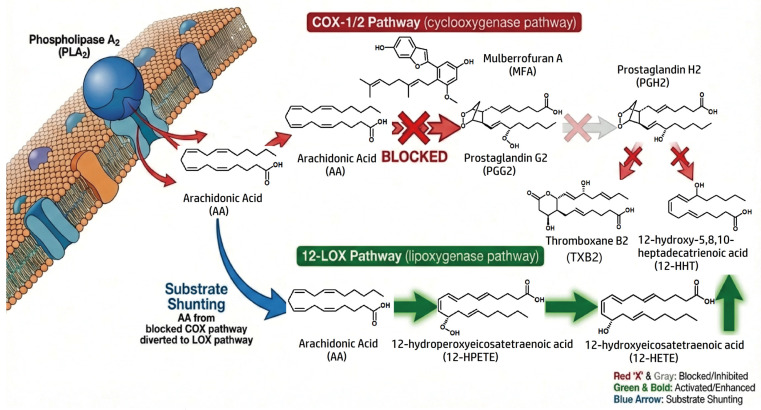
Proposed substrate-shunting model for MFA-associated modulation of arachidonic acid metabolism.

As shown in Figure 1, a substrate-shunting mechanism has been proposed as a plausible explanation for the simultaneous reduction in COX-derived metabolites and increase in 12-LOX-derived 12-HETE. In cellular systems, COX and LOX enzymes may compete for free AA as a common substrate. If the COX pathway is inhibited, a larger pool of AA may become available for metabolism through the LOX pathway, thereby increasing 12-HETE production. This mechanism would explain the apparent bidirectional metabolite pattern without requiring direct activation of 12-LOX by MFA.

However, this interpretation remains hypothetical unless supported by additional experiments [1,3]. For example, direct enzyme assays are needed to determine whether MFA inhibits COX isoforms selectively or affects LOX enzymes directly. Isotope-tracing studies, substrate-rescue experiments, selective COX/LOX inhibitors, and time-course metabolomic analyses would also help clarify whether the observed 12-HETE increase is truly caused by substrate redistribution. At present, the substrate-shunting model should be considered a testable mechanistic hypothesis rather than a confirmed mode of action.

MFA has been reported to reduce COX-associated metabolites such as HHT and TXB2 while increasing the 12-LOX-associated metabolite 12-HETE in platelet-based assays. A substrate-shunting model may explain this pattern, in which inhibition of the COX branch increases the availability of arachidonic acid for metabolism through the LOX branch. This model remains hypothetical and requires validation by direct enzyme assays, isotope-tracing experiments, and selective pathway inhibition studies.

### 4.3. Interpretive Limitations and Future Validation

As summarized in Table 2, this AA-related evidence represents one of the limited direct biological observations reported for MFA, but the current dataset remains insufficient to define its pharmacological significance [1,3]. Several limitations should be considered when interpreting the AA-related effects of MFA. First, the evidence appears to be based on a limited number of experimental systems, and independent replication remains insufficient. Second, the available data do not fully define dose-response relationships, COX isoform selectivity, LOX isoform involvement, or the dependence of the effect on platelet activation state. Third, platelet-based metabolite profiles cannot be directly extrapolated to systemic anti-inflammatory, antithrombotic, or cardiovascular outcomes without validation in cellular, animal, and eventually clinical models [25,27,33,34].

Future studies should evaluate purified MFA using standardized AA metabolism assays and appropriate positive controls, such as aspirin, indomethacin, or selective COX/LOX inhibitors. Quantitative parameters, including IC50 values for COX-related endpoints, EC50 or fold-change values for LOX-derived metabolites, and exact statistical reporting, should be provided. Such studies would clarify whether MFA is a selective modulator of AA metabolic flux, a conventional COX-pathway inhibitor, or a compound with more complex context-dependent effects [8].

## 5. Antimicrobial Activity: Direct Observations and Mechanistic Uncertainty

Antimicrobial activity is among the limited biological properties that have been reported directly for Mulberrofuran A (MFA) [3,6]. Available studies suggest that MFA may exhibit selective antibacterial activity, particularly against Gram-positive bacteria, whereas activity against Gram-negative organisms appears weak or absent under the tested conditions [3]. However, the compound-specific evidence base remains limited, and mechanistic interpretations are often extrapolated from broader studies on prenylated Morus phenolics rather than being demonstrated directly for MFA itself [1,7,8].

### 5.1. Reported Antibacterial Profile of MFA

Published reports indicate that MFA shows a relatively narrow antibacterial spectrum, with stronger activity against Gram-positive bacteria and mycobacterial species than against Gram-negative bacteria [3]. In contrast, little or no activity has been reported against Escherichia coli under some tested conditions, suggesting that bacterial envelope architecture may influence susceptibility. The available minimum inhibitory concentration (MIC) values are summarized in Table 3. As shown in Table 2, direct MFA-specific antimicrobial evidence remains limited, and much of the available interpretive context comes from related Morus constituents or scaffold-level comparisons [1,7,33].

### 5.2. Possible Structural Basis and Indirect Mechanistic Interpretation

The selective antibacterial profile of MFA may be partially related to its amphiphilic structural features, including phenolic hydroxyl groups and a lipophilic isoprenoid side chain [6,8]. From a physicochemical perspective, such a combination may facilitate interaction with lipid-rich membrane environments or membrane-associated proteins. This has led to the hypothesis that prenylated Morus phenolics, including MFA, may affect bacterial membrane integrity more readily in Gram-positive organisms, whose cell envelope lacks the outer membrane barrier characteristic of Gram-negative bacteria [3].

However, direct mechanistic confirmation for MFA remains insufficient. Much of the membrane-disruption interpretation is inferred from studies on structurally related prenylated flavonoids, stilbenoids, or other Morus-derived phenolics rather than from direct biophysical or ultrastructural experiments performed with MFA itself [7,12,33]. Therefore, proposed mechanisms such as membrane perturbation, pore formation, membrane depolarization, or leakage of intracellular contents should currently be regarded as plausible but unconfirmed explanations for the observed antibacterial pattern [1,3].

### 5.3. Current Limitations and Research Needs

Several limitations prevent definitive interpretation of MFA’s antimicrobial potential. First, the number of direct MFA-specific antimicrobial studies is limited [1,3]. Second, the currently available reports do not consistently provide standardized information on strain selection, biological replicates, bactericidal versus bacteriostatic distinction, time-kill kinetics, or statistical treatment of MIC determinations. Third, mechanistic conclusions regarding membrane disruption have not been validated by dedicated assays such as membrane permeability analysis, membrane potential measurements, electron microscopy, lipid bilayer interaction studies, or intracellular leakage assays.

Future work should evaluate purified MFA against a well-defined panel of Gram-positive, Gram-negative, and mycobacterial strains using harmonized antimicrobial protocols. In addition to MIC and minimum bactericidal concentration (MBC) values, studies should include time-kill analysis, synergy testing with standard antibiotics, and mechanistic experiments capable of distinguishing membrane-active effects from intracellular target inhibition. Such data would clarify whether MFA is best understood as a narrow-spectrum antibacterial lead, a scaffold for optimization, or simply a phytochemical with modest and context-dependent antimicrobial relevance [1,7,8].

## 6. Antiviral Relevance: Computational and Analogue-Based Evidence

Compared with the antibacterial observations reported for MFA, the antiviral relevance of MFA is currently supported mainly by computational and indirect evidence [14,34]. Recent molecular docking studies have suggested that MFA, or a closely related mulberrofuran compound depending on compound assignment, may interact with viral enzymatic targets such as 3C protease (3Cpro) [14,34]. However, no definitive conclusion regarding antiviral efficacy can be drawn without biochemical enzyme inhibition assays, cell-based antiviral assays, and selectivity evaluation [1,24].

### 6.1. Computational Prediction of Viral Target Interaction

Enterovirus 3C protease is an essential cysteine protease required for the cleavage of viral polyprotein precursors into functional structural and non-structural proteins. Because proteolytic processing is indispensable for viral replication, 3Cpro is widely considered an attractive antiviral target for enteroviruses such as EV-A71, EV-D68, coxsackieviruses, and poliovirus [34,35]. Computational studies have therefore explored natural products, including mulberrofuran-type compounds, as potential binders of 3Cpro or other viral proteins [14].

In this context, docking-based evidence suggests that MFA may form favorable interactions within the active-site region of viral 3Cpro [14]. Such findings are useful for target hypothesis generation, especially when supported by binding-energy calculations or molecular dynamics simulations. Nevertheless, docking scores alone do not establish target inhibition or antiviral activity. The predicted interaction must be confirmed using purified 3Cpro enzymatic assays, viral replication assays in infected cells, and cytotoxicity-adjusted selectivity indices [14,34].

### 6.2. Indirect Evidence from Other Morus Antiviral Constituents

Additional antiviral relevance can be inferred only indirectly from other Morus-derived constituents [1,16]. For example, certain mulberry compounds have been reported to interfere with enteroviral processes through mechanisms distinct from 3Cpro inhibition, such as interactions with viral capsid proteins involved in uncoating [24]. Mulberroside-type compounds and other stilbenoid derivatives provide useful contextual evidence that Morus metabolites can affect viral life-cycle events [16]. However, these compounds differ substantially from MFA in structure, polarity, metabolism, and likely target engagement [1].

Among mulberrofuran analogues, Mulberrofuran G has been reported to exhibit anti-hepatitis B virus activity and has also been investigated for interference with SARS-CoV-2 spike receptor-binding domain and ACE2 interaction [15]. These findings suggest that some members of the mulberrofuran family may have antiviral relevance [15,23]. However, Mulberrofuran G is structurally distinct from MFA and should be regarded as indirect analogue evidence rather than direct support for MFA-specific antiviral activity [1,23].

### 6.3. Current Limitations and Validation Requirements

The evidence map in Table 2 highlights that antiviral support for MFA remains largely Level II or Level III evidence. The antiviral potential of MFA remains preliminary. At present, the available evidence is insufficient to define its antiviral spectrum, potency, selectivity, or mechanism of action [14,34]. In particular, there is a need to verify whether MFA directly inhibits viral 3Cpro, whether the predicted binding translates into reduced viral replication, and whether any antiviral effect occurs at non-cytotoxic concentrations [14,24].

Future studies should include biochemical 3Cpro inhibition assays with appropriate positive controls, cell-based infection models for EV-A71, EV-D68, or other relevant viruses, time-of-addition experiments, viral load quantification, and cytotoxicity-adjusted selectivity index calculation [34,35]. If 3Cpro inhibition is proposed, resistance-mutation analysis or target-engagement experiments would further strengthen mechanistic interpretation. Until such data are available, MFA should be regarded as a computationally suggested antiviral candidate rather than an experimentally validated antiviral agent [1,14].

## 7. Metabolic Regulation: Indirect Enzyme-Inhibition Evidence and Hypothesis-Generating Relevance

The possible relevance of MFA to metabolic disorders, including diabetes and hyperlipidemia, has been proposed mainly on the basis of enzyme-inhibition studies involving related Morus constituents, structurally similar arylbenzofurans, and broader phytochemical evidence [1,11,12]. At present, direct MFA-specific evidence for glucose-lowering, insulin-sensitizing, or lipid-lowering effects remains insufficient [1,8]. Therefore, the metabolic implications of MFA should be considered hypothesis-generating rather than established pharmacological activities. As summarized in Table 2, most metabolic evidence relevant to MFA is Level II or Level III evidence derived from related arylbenzofurans, Morus extracts, or broader enzyme-inhibition studies [8,10,11,12].

### 7.1. α-Glucosidase and Postprandial Glucose Control: Evidence from Morus Constituents

Inhibition of intestinal α-glucosidase is a validated strategy for reducing postprandial glucose excursions by delaying carbohydrate digestion and glucose absorption. Several Morus-derived phenolic compounds, including stilbenoids and related polyphenols, have been reported to inhibit α-glucosidase in vitro [10,11,12]. These findings provide a useful rationale for investigating whether MFA or related arylbenzofurans might also affect carbohydrate-digesting enzymes [1,10].

However, the available evidence should not be interpreted as definitive proof that MFA itself acts as an α-glucosidase inhibitor unless purified MFA has been directly tested under standardized enzymatic conditions [11,12]. Future studies should determine the inhibitory potency of purified MFA against α-glucosidase, report IC50 values, compare its activity with acarbose or other positive controls, and assess inhibition kinetics. Without such data, α-glucosidase inhibition remains a plausible but unconfirmed mechanism for MFA.

### 7.2. PTP1B Inhibition and Insulin Signaling: Scaffold-Level Evidence

Protein tyrosine phosphatase 1B (PTP1B) is a negative regulator of insulin receptor signaling and has long been considered a potential therapeutic target for type 2 diabetes and obesity. Some arylbenzofuran derivatives and Morus-related phenolic scaffolds have been reported to inhibit PTP1B in vitro [8,11], suggesting that this structural class may be relevant to insulin-sensitizing mechanisms.

Nevertheless, the role of MFA in PTP1B inhibition remains to be clarified. If MFA has not been directly evaluated in purified PTP1B assays or insulin-responsive cellular systems, it should not be described as a confirmed PTP1B inhibitor. Even when in vitro enzyme inhibition is observed, further studies are needed to determine selectivity against related phosphatases, cellular target engagement, effects on insulin receptor substrate phosphorylation, glucose uptake, and cytotoxicity [1,11]. Therefore, PTP1B-related discussion should be framed as scaffold-level support for future testing rather than established evidence of MFA-mediated insulin sensitization.

### 7.3. Lipid Metabolism and LDL Oxidation: Analogue and Contextual Evidence

The potential relevance of MFA to lipid metabolism is even less directly established. Some related Morus constituents and mulberrofuran analogues have been associated with antioxidant, anti-atherogenic, or lipid-related effects, including inhibition of LDL oxidation or modulation of vascular risk factors [9,16,33]. These findings may be relevant because oxidative modification of LDL contributes to atherogenesis and because polyphenolic compounds can sometimes interfere with lipid peroxidation processes [9,16].

However, direct in vivo hypolipidemic data for MFA are currently lacking. Evidence obtained from Mulberrofuran G, Morus extracts, or other polyphenolic constituents cannot be directly extrapolated to MFA, especially because structural differences may alter absorption, metabolism, redox behavior, and biological target interactions [1,9]. Therefore, any proposed role of MFA in lipid regulation or atheroprotection should be regarded as an untested hypothesis.

### 7.4. Current Limitations and Experimental Priorities

The metabolic evidence related to MFA is limited by several factors. First, many studies involve crude Morus extracts or structurally related compounds rather than purified MFA [1,11]. Second, enzyme inhibition data, where available, may not translate into cellular or in vivo metabolic effects. Third, key parameters such as dose-response relationships, selectivity, bioavailability, and toxicity are often incompletely reported [1].

Future studies should evaluate purified MFA in standardized α-glucosidase and PTP1B inhibition assays, followed by validation in insulin-responsive cell models such as adipocytes, hepatocytes, or skeletal muscle cells [8,10,11]. Important endpoints should include IC50 values, inhibition kinetics, enzyme selectivity, insulin-stimulated phosphorylation of insulin receptor signaling components, GLUT4 translocation, glucose uptake, and cytotoxicity. For lipid-related hypotheses, MFA should be tested in LDL oxidation assays, foam-cell formation models, and appropriate animal models of dyslipidemia or atherosclerosis [9,16,33]. These studies are necessary before MFA can be considered a credible candidate for metabolic disease intervention.

## 8. Cardiovascular-Related Evidence: Platelet, Vascular, and Analogue-Based Clues

The cardiovascular relevance of MFA remains largely inferential. While *Morus alba* root bark and several Morus-derived phenolic constituents have been associated with hypotensive, vasorelaxant, anti-inflammatory, or anti-atherogenic effects [1,13,26,33], direct in vivo cardiovascular data for purified MFA are currently lacking. Therefore, cardiovascular implications for MFA should be considered preliminary and hypothesis-generating, based mainly on platelet-related observations, vascular effects of Morus extracts, and pharmacological data from structurally related compounds [1,26,33]. As indicated in Table 2, the cardiovascular evidence relevant to MFA consists mainly of Level II analogue-based data and Level III extract-based data, with limited direct MFA-specific validation [1].

### 8.1. Hypotensive and Vasorelaxant Evidence from Morus Extracts and Related Analogues

Several Morus-derived constituents have been investigated for vascular effects. For example, Mulberrofuran F and Mulberrofuran G were reported to produce depressor responses in rabbit intravenous injection models, with blood pressure reductions reported after administration of low doses [13]. In addition, Morus root bark extracts and certain Diels–Alder-type adducts, including kuwanon-type compounds, have been associated with vasorelaxant or antihypertensive effects, in some cases involving endothelium-dependent mechanisms [26,33].

These findings indicate that the Morus root bark phytochemical space contains compounds with vascular bioactivity [1,34]. However, they do not establish antihypertensive activity for MFA. Mulberrofuran F, Mulberrofuran G, and kuwanon-type adducts differ structurally from MFA and may differ in pharmacokinetics, target engagement, and vascular mechanism [13]. Therefore, MFA’s potential involvement in blood-pressure regulation remains speculative until tested directly in vascular preparations and in vivo cardiovascular models [1,34].

### 8.2. Platelet and Arachidonic Acid-Related Clues Relevant to Vascular Biology

As discussed above, MFA has been reported to affect arachidonic acid-derived metabolites in platelet-based systems, including a reduction in COX-associated metabolites such as TXB2 [3]. Because thromboxane signaling is relevant to platelet activation and vascular tone, these observations provide a mechanistic rationale for further investigation of MFA in platelet and vascular models [34].

However, platelet metabolite modulation should not be equated with demonstrated antithrombotic or cardioprotective efficacy [26,33]. The simultaneous increase in 12-HETE reported in the same experimental context further complicates interpretation, as 12-HETE can participate in inflammatory and vascular signaling [25,33]. Thus, the vascular implications of MFA’s AA-related profile remain uncertain and require direct functional validation, including platelet aggregation assays, vascular contraction/relaxation studies, and in vivo thrombosis or hypertension models [26,33].

### 8.3. Inflammatory Signaling: Indirect Evidence from Mulberrofuran K and Morus Extracts

Inflammatory signaling is closely linked to vascular dysfunction and atherosclerosis. Some evidence relevant to this area comes from structurally related mulberrofuran analogues rather than MFA itself. In particular, Mulberrofuran K has been reported to reduce inflammatory mediators such as nitric oxide, reactive oxygen species, IL-1β, IL-6, and TNF-α in LPS-stimulated RAW264.7 macrophages [25]. These effects were associated with suppression of NF-κB and ERK1/2 signaling and downregulation of inflammatory enzymes such as iNOS and COX-2 [25,27].

These findings suggest that certain mulberrofuran analogues may modulate inflammatory signaling pathways [25]. Nevertheless, they should not be interpreted as direct evidence that MFA exerts the same transcriptional effects. The structural differences between MFA and Mulberrofuran K may influence cellular uptake, potency, selectivity, and pathway engagement [25]. Therefore, the proposed anti-inflammatory relevance of MFA requires direct testing in macrophages, endothelial cells, vascular smooth muscle cells, and appropriate inflammatory vascular models [13,34].

### 8.4. Limitations and Future Cardiovascular Validation

Current cardiovascular interpretation is limited by the scarcity of MFA-specific studies [1]. Most available evidence comes from Morus extracts, Diels–Alder-type adducts, or related mulberrofuran analogues [1,13,16]. In addition, many reported vascular or anti-inflammatory effects have not been linked to compound-specific exposure, pharmacokinetics, or target engagement [34].

Future studies should evaluate purified MFA in platelet aggregation assays, endothelial nitric oxide production assays, vascular ring experiments, and cellular models of endothelial inflammation and smooth muscle proliferation [26,33]. In vivo studies should assess blood pressure, thrombosis tendency, vascular reactivity, inflammatory biomarkers, and safety parameters after defined MFA administration. Without such evidence, MFA should not yet be described as a cardioprotective or antihypertensive agent, but rather as a compound with mechanistic clues warranting cardiovascular investigation [1,26,33].

## 9. Oncology-Related Evidence: Computational Predictions and Analogue-Based Mechanistic Clues

As summarized in Table 2, oncology-related support for MFA is dominated by Level II analogue-based evidence and Level III scaffold-level or computational evidence, with limited direct MFA-specific validation [36,37]. The potential relevance of MFA to oncology remains largely indirect. Although several Morus-derived phenolics, including morusin, kuwanon-type compounds, and mulberrofuran analogues, have been associated with antiproliferative or pro-apoptotic effects [18,19], direct experimental evidence demonstrating antitumor activity of purified MFA is currently limited [1]. Therefore, MFA should not yet be described as an established anticancer compound. Instead, the available literature provides a set of computational predictions and analogue-based mechanistic clues that may guide future experimental testing [36,37].

### 9.1. Aromatase Docking and Breast Cancer-Related Hypotheses

One proposed oncology-related direction for MFA concerns estrogen-dependent breast cancer. In silico molecular docking studies have predicted that MFA may bind to aromatase (CYP19A1), the enzyme responsible for the conversion of androgens to estrogens [22,38]. Because aromatase is a clinically validated target in hormone receptor-positive breast cancer, this computational finding provides a rationale for further testing MFA in aromatase-related assays.

However, docking evidence alone cannot establish MFA as an aromatase inhibitor or breast cancer therapeutic candidate. Binding-energy estimates are sensitive to docking protocol, protein conformation, scoring function, and comparator selection. In particular, if lapatinib was used as a comparator in the original docking study, this should be interpreted cautiously because lapatinib is an EGFR/HER2 tyrosine kinase inhibitor rather than a standard aromatase inhibitor. Future studies should compare MFA with established aromatase inhibitors such as letrozole, anastrozole, or exemestane, and should include biochemical aromatase inhibition assays, estrogen-production assays, and estrogen-dependent proliferation models in ER-positive breast cancer cells [22,38].

### 9.2. JAK2/STAT3 and Apoptosis-Related Evidence from Mulberrofuran G

A second line of indirect evidence comes from studies on Mulberrofuran G, a structurally related but more complex Morus-derived compound. Mulberrofuran G has been reported to induce apoptosis in leukemia cells and to suppress cancer cell proliferation or migration in association with reduced phosphorylation of JAK2 and STAT3 and altered expression of downstream targets such as Cyclin D1 and MMP-2 [23,36]. These findings suggest that certain mulberrofuran analogues may influence oncogenic signaling pathways.

Nevertheless, these observations should not be directly attributed to MFA. Mulberrofuran G differs from MFA in molecular size, stereochemical complexity, substituent pattern, and potentially in cellular uptake and target engagement [1,23]. Therefore, the JAK2/STAT3-related anticancer mechanism should be treated as analogue-based evidence that supports future testing of MFA, rather than as evidence that MFA itself has anti-metastatic activity.

### 9.3. UPR, ER Stress, and Other 2-Arylbenzofuran Scaffold-Level Evidence

Beyond Morus-specific analogues, some 2-arylbenzofuran derivatives have been reported to induce endoplasmic reticulum stress and activation of the unfolded protein response in gastrointestinal cancer models [37]. Because sustained or severe ER stress can contribute to apoptotic cell death, this scaffold-level evidence suggests another possible mechanism by which arylbenzofuran-type compounds may affect cancer cell viability.

However, the extent to which these findings apply to MFA remains unknown. The biological consequences of ER stress are context-dependent and may involve adaptive survival responses as well as apoptosis. Therefore, UPR activation should not be assumed to represent a confirmed anticancer mechanism for MFA without direct testing in defined cancer cell models, accompanied by markers such as PERK/eIF2α, ATF4, CHOP, IRE1α/XBP1, caspase activation, and rescue experiments using ER-stress modulators [37].

### 9.4. GSK-3β Inhibition: Relevance and Oncological Ambiguity

Arylbenzofuran derivatives from *Morus alba* have also been investigated as inhibitors of glycogen synthase kinase-3β (GSK-3β), a kinase involved in multiple signaling networks, including glycogen metabolism, neuroinflammation, cell survival, and Wnt/β-catenin signaling [11,37]. This has prompted interest in whether MFA-related scaffolds might be relevant to cancer biology.

The oncological implications of GSK-3β inhibition are complex and tumor-context dependent. In some settings, GSK-3β inhibition may reduce cancer cell survival or inflammatory signaling. In other settings, particularly in tumors driven by aberrant Wnt/β-catenin activation, inhibition of GSK-3β may stabilize β-catenin and potentially enhance oncogenic transcription. Therefore, GSK-3β inhibition should not be presented as uniformly anticancer. Any claim regarding MFA, GSK-3β, and tumor suppression requires direct evidence in pathway-defined cancer models and careful assessment of β-catenin-dependent transcriptional outcomes [11,37].

### 9.5. Limitations and Future Oncology Validation

At present, the oncology-related evidence for MFA is insufficient to define its anticancer potency, selectivity, or mechanism of action [1,37]. Much of the available support comes from computational docking, structurally related mulberrofuran analogues, other Morus phenolics, or broader 2-arylbenzofuran scaffold studies [22,36,37,38]. These lines of evidence are useful for hypothesis generation but cannot substitute for compound-specific experimental validation.

Future studies should evaluate purified MFA across a panel of cancer and non-cancer cell lines, with attention to dose-response relationships, cytotoxicity selectivity, apoptosis versus cytostasis, and mechanism-specific endpoints. For aromatase-related hypotheses, biochemical CYP19A1 inhibition and ER-positive breast cancer models are required [22,38]. For JAK2/STAT3 hypotheses, phosphorylation assays, target rescue experiments, and migration/invasion assays should be performed [23,36]. For GSK-3β-related hypotheses, kinase inhibition assays, pathway selectivity profiling, and β-catenin transcriptional readouts are essential [11,37]. Until such evidence is available, MFA should be considered an oncology-relevant candidate for future testing rather than a validated antitumor agent [1,37].

## 10. Antioxidant and Immunomodulatory Relevance: Structural Rationale and Indirect Evidence

As summarized in Table 2, antioxidant and immunomodulatory support for MFA is primarily Level II or Level III evidence, derived from related mulberrofuran analogues or complex extract studies. The antioxidant and immunomodulatory relevance of MFA is plausible from a chemical perspective, given its phenolic structure and its relationship to bioactive Morus-derived polyphenols [1,31]. However, direct MFA-specific evidence remains limited. Much of the current discussion is based on structurally related mulberrofuran analogues, Morus extracts, or broader phytochemical studies [9,25,27]. Therefore, antioxidant and immunomodulatory effects should be presented as possible areas for future investigation rather than as established pharmacological properties of MFA [1].

### 10.1. Antioxidant Potential: Phenolic Chemistry and Analogue-Based Evidence

Phenolic hydroxyl groups can contribute to redox-related activity through hydrogen donation, radical stabilization, metal-chelating interactions, or modulation of oxidative signaling pathways. On this basis, MFA may be expected to possess some antioxidant potential. Nevertheless, structural expectation alone is insufficient to define antioxidant potency.

Experimental antioxidant data are more clearly available for related mulberrofuran analogues. For example, Mulberrofuran B has been reported to show stronger antioxidant activity than morusin in several in vitro assays, including phosphomolybdenum (1531.33 ± 20.28 mmol/g), ferricyanide reducing power (14.39%), ABTS (IC50 = 95.74 ± 4.21 μM), and DPPH radical-scavenging tests (IC50 = 843.87 ± 10.65 μM) [9]. Mulberrofuran C has also been reported to exhibit strong antioxidant activity in Trolox-comparative assays. These findings suggest that antioxidant capacity may be a relevant property within the mulberrofuran family [9,31].

However, the antioxidant potency of MFA itself cannot be assumed from these analogues. Differences in hydroxylation pattern, prenylation or geranylation, conformational flexibility, and solubility can strongly influence antioxidant assay outcomes. Moreover, chemical radical-scavenging assays do not necessarily predict intracellular antioxidant effects or in vivo redox modulation. Therefore, MFA should be directly evaluated using both chemical assays and cellular oxidative-stress models before antioxidant claims are made [9,31]. Moreover, DPPH, ABTS, TEAC, ferricyanide reducing power, and phosphomolybdenum assays differ in reaction mechanism and assay conditions; therefore, quantitative comparison should be made cautiously and preferably within the same standardized assay system [39].

### 10.2. Immunomodulatory Relevance: Limited and Contextual Evidence

Immunomodulation is broader than anti-inflammatory activity and includes direct or indirect regulation of immune-cell activation, cytokine production, antigen presentation, macrophage polarization, lymphocyte function, and innate immune signaling. At present, the evidence supporting immunomodulatory activity of MFA is limited and largely contextual [25,27].

Some phytochemical or network-based studies have reported MFA as a putative constituent or bioactive marker in complex herbal extracts and have linked such extracts to immunomodulatory or redox-related functions [1]. However, when MFA is identified within a complex mixture, the observed biological activity cannot be attributed to MFA without isolation, quantification, and compound-specific testing. Similarly, studies performed in immune-related cell lines such as THP-1 or RAW264.7 using related compounds, extracts, or analogues provide useful context but do not establish direct immunomodulatory activity for MFA [25,27].

### 10.3. Limitations and Future Validation

Several issues limit current interpretation. First, direct antioxidant and immunomodulatory studies using purified MFA are scarce [1]. Second, evidence from Mulberrofuran B, Mulberrofuran C, Mulberrofuran K, or complex plant extracts should be treated as indirect [9,12,31]. Third, antioxidant assays such as DPPH and ABTS may reflect chemical reducing capacity rather than biologically meaningful redox regulation [9,31]. Fourth, immunomodulatory claims require careful distinction between nonspecific cytotoxicity, general anti-inflammatory suppression, and true immune regulation [25,27].

Future studies should evaluate purified MFA in standardized antioxidant assays and cellular oxidative-stress models, including ROS generation, antioxidant enzyme expression, mitochondrial oxidative stress, and cell viability controls [9,31]. Immunomodulatory studies should include macrophage, monocyte, dendritic-cell, and lymphocyte models, with endpoints such as cytokine profiles, NF-κB/MAPK activation, inflammasome signaling, macrophage polarization markers, and immune-cell viability [25,27]. Until these studies are performed, MFA should be considered a chemically plausible but insufficiently validated antioxidant and immunomodulatory candidate [1].

## 11. Metabolic Fate and Pharmacokinetic Considerations: Structural Inference and Evidence Gaps

The pharmacokinetic behavior of Mulberrofuran A (MFA) has not been systematically characterized. To date, dedicated studies defining its absorption, distribution, metabolism, excretion, plasma exposure, tissue distribution, half-life, or bioavailability are lacking [1,16]. Therefore, current discussion of MFA pharmacokinetics must rely largely on structural inference and comparison with related Morus constituents or 2-arylbenzofuran-type compounds [16,29]. Such comparisons are useful for hypothesis generation but should not be interpreted as direct pharmacokinetic evidence for MFA [1].

### 11.1. Structural Features Relevant to Absorption and Membrane Permeability

MFA is a non-glycosylated arylbenzofuran containing phenolic hydroxyl groups and an isoprenoid side chain. Compared with hydrophilic stilbene glycosides such as Mulberroside A, MFA is expected to be more lipophilic [16]. This structural difference may favor passive interaction with lipid membranes and could potentially facilitate intestinal epithelial permeability.

However, higher lipophilicity does not necessarily guarantee higher oral bioavailability. Solubility, dissolution rate, intestinal efflux transporters, plasma protein binding, first-pass metabolism, and chemical stability may all influence systemic exposure. Therefore, while the non-glycosylated and lipophilic nature of MFA provides a plausible basis for investigating intestinal absorption, claims of superior absorption kinetics remain speculative until supported by Caco-2 permeability assays, in situ intestinal perfusion, or in vivo pharmacokinetic studies [1,16].

### 11.2. Gastrointestinal Stability and Possible Microbial Transformation

The gastrointestinal fate of MFA is also insufficiently defined. In vitro colon fermentation and anaerobic incubation studies of selected 2-arylbenzofurans suggest that this scaffold may undergo microbial transformation under colonic conditions [29]. Possible reactions for phenolic benzofuran-type compounds may include reduction, demethylation, dehydroxylation, side-chain modification, or ring-related transformation, depending on the exact structure and microbial community [29].

Nevertheless, direct evidence for microbial metabolism of MFA remains limited. Therefore, it should not be assumed that MFA follows a defined microbial pathway or that its metabolites retain biological activity. Future studies should use purified MFA in simulated gastric, intestinal, and colonic systems, combined with LC–MS/MS or high-resolution metabolomics, to identify specific degradation products and microbial metabolites [1,29].

### 11.3. Phase II Metabolism and First-Pass Considerations

Because MFA contains phenolic hydroxyl groups, it is reasonable to hypothesize that it may undergo Phase II conjugation, particularly glucuronidation and sulfation, after intestinal or hepatic exposure. Such metabolism is common for polyphenolic natural products and may reduce the circulating concentration of the unconjugated parent compound while increasing the abundance of hydrophilic conjugates [16].

**Figure 2:**
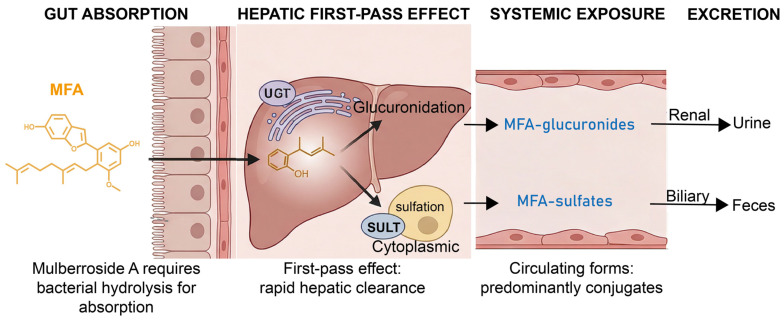
Proposed pharmacokinetic framework for Mulberrofuran A based on structural inference and related-compound evidence.

However, this remains an inference rather than an experimentally established pharmacokinetic profile for MFA. The specific UGT or SULT isoforms involved, metabolic rates, conjugate structures, plasma exposure, biliary versus urinary excretion, and potential enterohepatic recycling have not been clearly defined (Figure 2). Therefore, statements regarding rapid clearance, short parent-compound half-life, or dominance of MFA-glucuronides and MFA-sulfates in plasma should be framed as testable hypotheses. Dedicated liver microsome, hepatocyte, intestinal S9, and in vivo pharmacokinetic studies are needed to confirm these possibilities [1,16].

### 11.4. Future Pharmacokinetic Priorities

Future pharmacokinetic studies should evaluate purified MFA using validated analytical methods. Key parameters should include aqueous solubility, chemical and gastrointestinal stability, intestinal permeability, plasma protein binding, metabolic stability in liver and intestinal microsomes, metabolite identification, oral and intravenous pharmacokinetics, tissue distribution, and excretion routes. Quantitative parameters such as Cmax, Tmax, AUC, half-life, clearance, volume of distribution, and absolute oral bioavailability are essential for assessing whether the in vitro bioactivity of MFA can translate into systemic or local in vivo effects [1,16,29].

Overall, MFA has structural features that may influence absorption and metabolism, but its pharmacokinetic profile remains largely undefined [1]. Current discussion should therefore be limited to plausible structural considerations and should avoid describing MFA as having confirmed superior absorption, defined gut stability, or established rapid Phase II clearance [16,29].

This model summarizes plausible absorption and metabolic routes for MFA based on its non-glycosylated arylbenzofuran structure, phenolic hydroxyl groups, and lipophilic isoprenoid side chain. Possible processes include intestinal absorption, microbial transformation, and Phase II conjugation through glucuronidation or sulfation. These pathways remain hypothetical for MFA and require direct validation using purified compound pharmacokinetic and metabolomic studies.

## 12. Safety and Toxicity: Limited Direct Evidence and Theoretical Considerations

The safety profile of purified MFA has not been adequately defined. Although Morus species have a long history of dietary and medicinal use, the safety of whole plant materials or traditional preparations cannot be directly equated with the safety of isolated MFA, especially at concentrated pharmacological doses [1]. Therefore, safety discussion should distinguish traditional exposure to complex Morus matrices from compound-specific toxicological evaluation [1].

### 12.1. Natural Occurrence and Limits of Dietary Safety Inference

Mulberry fruits, leaves, and root bark have been used in food and traditional medicine, suggesting that Morus-derived preparations may be tolerated under customary exposure conditions [1,32]. However, MFA is only one constituent within a complex phytochemical matrix, and its concentration, bioavailability, and metabolic fate may differ substantially between traditional preparations and purified compound administration [1,16].

Consequently, natural origin should not be used as evidence of compound-level safety. Isolated MFA may produce different exposure levels, tissue distribution, or metabolic burdens than those encountered during dietary or traditional use. Direct toxicological studies are therefore required before MFA can be considered safe as a purified pharmacological agent [1].

### 12.2. Benzofuran Scaffold and Theoretical Toxicological Concerns

The benzofuran scaffold is present in diverse natural and synthetic compounds with variable safety profiles. Some synthetic benzofuran derivatives have been associated with hepatic, neurological, or other toxicological liabilities, depending on their substitution pattern, metabolic activation, and target interactions [26]. These concerns do not imply that MFA is intrinsically toxic, but they justify careful toxicological evaluation.

For phenolic benzofuran-type compounds, possible safety-related issues include oxidative metabolism, formation of reactive intermediates, off-target enzyme interactions, mitochondrial stress, or interference with drug-metabolizing enzymes and transporters [26,29]. At the same time, phenolic hydroxyl groups may facilitate detoxification through glucuronidation or sulfation. The net toxicological outcome therefore cannot be predicted confidently from structure alone and must be experimentally determined [1,26].

### 12.3. Cytotoxicity, Selectivity, and Therapeutic Index

Because MFA has been discussed in relation to antimicrobial and oncology-related applications, cytotoxic selectivity is an important unresolved issue [7,37]. Any activity against bacteria or cancer cells must be interpreted alongside effects on non-malignant mammalian cells. Without parallel cytotoxicity testing, selectivity index calculation, and exposure-response analysis, it is not possible to determine whether observed bioactivity reflects useful selectivity or nonspecific cellular toxicity [7,18,19].

Future studies should therefore include viability assays in non-transformed human cell lines, hemolysis assays, mitochondrial toxicity assays, and comparisons between active concentrations and cytotoxic concentrations. For antimicrobial testing, MIC and MBC values should be accompanied by mammalian cytotoxicity data. For anticancer testing, tumor-cell inhibition should be compared with toxicity toward matched normal or non-transformed cells [7,37].

### 12.4. Required Toxicological Studies

Direct in vivo toxicity data for MFA remain limited [1]. Essential safety studies should include acute and repeated-dose toxicity, liver and kidney function assessment, hematological parameters, histopathology, genotoxicity, reproductive toxicity, and long-term exposure evaluation. In vitro safety studies should assess CYP inhibition or induction, UGT/SULT interactions, transporter effects, reactive metabolite formation, and potential drug–drug interaction risk [16,26,29].

Overall, current evidence is insufficient to conclude that MFA has a low toxicity profile [1]. Its natural origin and plausible metabolic conjugation may support cautious optimism, but they do not replace formal toxicological assessment [1,16]. Before MFA can be advanced as a purified therapeutic candidate, its safety margin, metabolic liabilities, and long-term toxicity must be established using standardized preclinical models [1].

## 13. Conclusions and Future Perspectives

This review summarizes the available evidence on Mulberrofuran A (MFA) and related Morus-derived congeners, with their structures and reported activities compiled in Table 4. A systematic comparison of in silico predictions and experimental evidence across major pharmacological targets, together with identified validation gaps, is provided in Table S2. Overall, the current literature suggests that subtle structural variations within the mulberrofuran and broader Morus phenolic family may influence biological activity. However, these relationships should be regarded as preliminary structure–activity trends rather than established SAR rules, because many reported activities are derived from different compounds, assay systems, extracts, or computational models.

Several tentative structure-related observations can be drawn. First, monomeric 2-arylbenzofurans such as MFA may provide a compact phenolic scaffold with lipophilic substituents that could support membrane interaction and enzyme binding. Second, prenyl or geranyl side chains may influence lipophilicity, cellular permeability, and target engagement, although their exact contribution requires direct comparative testing. Third, phenolic hydroxylation patterns appear relevant to redox behavior and enzyme interaction, as suggested by antioxidant data from related analogues such as Mulberrofuran B and Mulberrofuran C. Fourth, glycosylation may substantially alter solubility, intestinal processing, and pharmacokinetic behavior, but direct comparison between MFA and glycosylated Morus constituents remains incomplete. Fifth, Diels–Alder-type adducts and larger polycyclic congeners may possess biological profiles distinct from monomeric arylbenzofurans, but their activities should not be directly extrapolated to MFA. Accordingly, the current evidence base should be interpreted as a hypothesis-generating framework rather than a definitive pharmacological validation of MFA.

Within this framework, MFA is best viewed as a chemically attractive and biologically plausible lead compound rather than as a fully validated multi-target therapeutic agent. The strongest direct evidence relates to limited antibacterial observations and reported modulation of arachidonic acid-derived metabolites in platelet-based systems. Other proposed activities, including antidiabetic, antiviral, antihypertensive, anticancer, immunomodulatory, and broader anti-inflammatory effects, remain largely supported by analogue-based evidence, extract-level observations, or computational predictions. Therefore, the therapeutic relevance of MFA is promising but experimentally underdeveloped.

Future research should prioritize direct compound-specific validation. Purified MFA should be evaluated in standardized biochemical, cellular, and in vivo models with appropriate positive controls, dose-response analysis, cytotoxicity assessment, and statistical reporting. Key hypotheses requiring confirmation include COX/LOX-related modulation, PTP1B and α-glucosidase inhibition, viral protease binding, aromatase inhibition, vascular effects, inflammatory signaling regulation, and cancer-related pathway modulation. Such studies are necessary to determine whether MFA itself, rather than related Morus constituents, is responsible for the proposed biological activities. Future in vivo studies should include adequate sample-size justification, predefined statistical analysis, and transparent reporting standards [40].

Equally important is the systematic evaluation of pharmacokinetics and safety. Current ADME and toxicological data for purified MFA remain insufficient, and inferences from Mulberroside A, Morus extracts, or generic benzofuran scaffolds cannot substitute for MFA-specific studies. Future work should define solubility, permeability, metabolic stability, Phase II conjugation, plasma exposure, tissue distribution, bioavailability, excretion, drug–drug interaction potential, and acute and chronic toxicity. Without these data, the translational potential of MFA cannot be reliably assessed.

In conclusion, MFA represents a valuable Morus-derived 2-arylbenzofuran scaffold with multiple testable pharmacological hypotheses. Its future significance will depend on rigorous experimental validation, transparent evidence grading, and integrated ADME–toxicity assessment. Bridging these gaps will determine whether MFA can progress from a phytochemical of mechanistic interest to a credible lead for therapeutic development.

## Figures and Tables

**Table 1:** Representative compounds related to MFA in *Morus alba* and their evidence relevance.

Compound	Chemical Class	Key Structural Features	Primary Source	Evidence Category	Reported Activity	Relevance to MFA	Key References
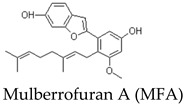	Monomeric benzofuran/isoprenoid-substituted 2-arylbenzofuran	Monomeric arylbenzofuran with an isoprenoid side chain and phenolic hydroxyl groups	Root Bark (Radix Mori)	Direct MFA-specific evidence	Structural identification; limited reported antimicrobial and arachidonic acid-related activity	Core compound of the present review and primary reference point for evaluating direct versus indirect evidence	[6]
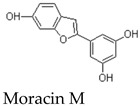	Monomeric Benzofuran	Non-isoprenylated benzofuran core	Leaves and Twigs	Structural comparator/indirect evidence	Primarily phytochemical and structural relevance; moracin-type compounds have been associated with multiple biological activities in broader Morus literature	Useful as a structural or biosynthetic reference scaffold, but not a substitute for MFA-specific evidence	[1,5,22]
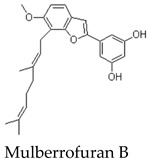	Isoprenylated 2-Arylbenzofuran	Closely related monomeric analogue of MFA with a different substitution pattern	Root Bark	Indirect evidence from related mulberrofuran analogue	Reported in phytochemical studies and antioxidant-related discussions involving mulberry root constituents	Useful for limited structure–activity comparison, but its activity cannot be directly extrapolated to MFA	[9,22]
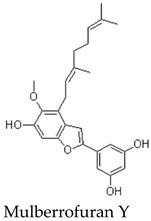	Isoprenylated/geranylated 2-arylbenzofuran	Monomeric arylbenzofuran with a geranyl-type side chain	Root Bark	Indirect evidence from related mulberrofuran analogue	Primarily reported as a phytochemical constituent; direct pharmacological characterization is limited	Relevant as a structural analogue with similar lipophilic substitution, but currently of limited functional interpretive value	[22]
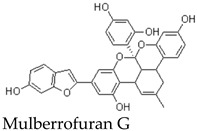	Diels–Alder type adduct	Complex polycyclic mulberrofuran-family adductsystem	Root Bark	Indirect evidence from related mulberrofuran analogue	Reported anti-hepatitis B virus activity and interference with SARS-CoV-2 spike RBD–ACE2 interaction	Provides indirect support for antiviral relevance within the mulberrofuran family, but is structurally more complex than MFA and cannot be treated as direct MFA evidence	[13,15,23]
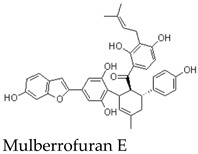	Diels–Alder type adduct	Rearranged and highly oxygenated complex adduct	Callus Culture	Structural/contextual evidence	Mainly relevant to phytochemical diversity and biosynthetic plasticity	Useful for illustrating structural diversity within the mulberrofuran family rather than inferring MFA bioactivity	[2,22]
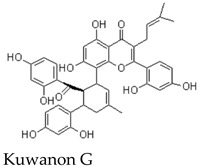	Diels–Alder type adduct/prenylated flavonoid–chalcone adduct	Isoprenylated Flavonoid-Chalcone adduct; potent enzyme inhibitor	Root Bark	Indirect evidence from related Morus compound	Reported enzyme inhibitory and other bioactivities in broader Morus phytochemical literature	Relevant as a comparator for the pharmacological richness of Morus phenolics, but not a direct MFA-related compound	[1,2]
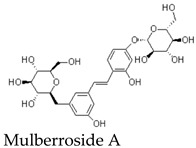	Stilbenoid Glycoside	Oxyresveratrol diglucoside; major water-soluble phenolic constituent	Root Bark and Twigs	Contextual evidence/related Morus constituent	Reported tyrosinase inhibition, metabolism, and broader antioxidant-related relevance in mulberry literature	Important as a contextual Morus constituent and comparator in pharmacokinetic discussion, but should be clearly distinguished from MFA	[16,24]
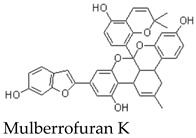	Mulberrofuran analogue	Structurally related mulberrofuran congener	Root bark	Indirect evidence from related mulberrofuran analogue	Reported anti-inflammatory activity in Morus bark-derived studies	Supports the possibility that some mulberrofuran analogues modulate inflammatory pathways, but does not establish equivalent activity for MFA	[25]

Note: “Evidence category” indicates the level of interpretive relevance to MFA. Direct MFA-specific evidence refers to studies in which purified Mulberrofuran A itself was isolated or tested. Indirect evidence refers to structurally related mulberrofuran analogues or other Morus-derived compounds used for comparative or hypothesis-generating purposes. Contextual evidence refers to compounds included primarily to illustrate phytochemical diversity, biosynthetic context, or broader pharmacological background. Reported activities listed for related compounds should not be interpreted as confirmed activities of MFA unless directly validated.

**Table 2:** Evidence map of studies relevant to Mulberrofuran A (MFA): evidence level, study type, main findings, and methodological limitations.

Reference	Tested Material	Evidence Level	Study Type	Model/ System	Main Finding	Quantitative/ Statistical Reporting	Relevance to MFA	Key Limitation
Nomura et al., 1978 [6]	Mulberrofuran A	Level I: Direct MFA-specific evidence	Phytochemical isolation/structural identification	Root bark of Morus alba	First isolation and structural characterization of Mulberrofuran A as an isoprenoid-substituted 2-arylbenzofuran	Structural study; no pharmacological statistical analysis	Foundational evidence establishing the chemical identity of MFA	No direct pharmacological testing; limited translational relevance by itself
Sureshan et al., 2024 [14]	“Mulberrofuran” compound (should be verified whether specifically MFA)	Level I or II depending on compound identity	In silico study	HAV 3Cpro and RdRP docking/molecular dynamics/DFT	Suggested potential inhibitory interaction with viral targets	Computational scores reported; no experimental validation or biological statistics	Potential antiviral relevance if the tested compound is confirmed as MFA	Purely computational; no biochemical, cellular, or in vivo confirmation
Martins et al., 2021 [9]	Mulberrofuran B and morusin in ethanol extract of *Morus alba* roots	Level II: Related analogue evidence	Antioxidant-related experimental study	Root extract/antioxidant assays	Suggested antioxidant contribution of mulberrofuran B and morusin in root extract context	Quantitative antioxidant-related data reported, but MFA not tested	Supports indirect structure-related discussion of mulberry benzofurans	Not MFA-specific; extract-associated context complicates attribution
Yang et al., 2011 [11]	Constituents from *Morus alba* var. multicaulis	Level III: Contextual extract/related constituent evidence	In vitro bioactivity study	3T3-L1 cells; RAW264.7 cells	Inhibitory effects on adipocyte differentiation and nitric oxide production	Experimental activity data reported; MFA not specifically tested	Context for anti-inflammatory and metabolic relevance of Morus-derived constituents	Multi-compound context; not MFA-specific
Geng et al., 2012 [23]	Mulberrofuran G and isomulberrofuran G	Level II: Related analogue evidence	Experimental antiviral/phytochemical study	Anti-HBV-related assays; MS fragmentation	Reported anti-hepatitis B virus activity of mulberrofuran G analogues	Quantitative antiviral assay data reported in analogue study	Indicates antiviral potential within the mulberrofuran family	Structurally related but not interchangeable with MFA
Panth et al., 2018 [26]	Ethanol extract of *Morus alba* root bark	Level III: Contextual extract evidence	In vitro/ex vivo vascular study	Rat aorta; smooth muscle cell migration and proliferation	Reported endothelium-dependent relaxation and vascular protective effects	Experimental data and statistics reported for extract	Provides background for cardiovascular relevance of root bark constituents	Crude extract; cannot attribute effects to MFA
Fukai et al., 1985 [13]	Mulberrofurans F and G	Level II: Related analogue evidence	Phytochemical/bioactivity study	Natural hypotensive compound characterization	Reported hypotensive Diels–Alder-type adducts from mulberry	Bioactivity context reported, but not MFA-specific	Supports family-level discussion of cardiovascular relevance	Different analogues with more complex structures than MFA
Shim et al., 2018 [25]	Mulberrofuran K	Level II: Related analogue evidence	In vitro anti-inflammatory study	Bark-derived compound tested in inflammatory models	Reported anti-inflammatory activity of mulberrofuran K	Experimental/statistical data reported for analogue	Suggests inflammatory relevance of some mulberrofuran analogues	Not MFA-specific; analogue activity cannot be directly transferred
Suriyaprom et al., 2023 [27]	White mulberry leaf extracts	Level III: Contextual extract evidence	In vitro antioxidant/anti-inflammatory study	LPS-stimulated RAW264.7 macrophages	Leaf extracts showed antioxidant and anti-inflammatory effects	Quantitative and statistical data reported for extracts	Contextual support for mulberry anti-inflammatory reputation	Extract study; no isolated MFA
Paudel et al., 2019 [28]	Arylbenzofurans from *Morus alba* root bark	Level II: Related scaffold evidence	In vitro + in silico enzyme inhibition study	Cholinesterase, BACE1, GSK-3β assays	Reported multi-target neurorelevant inhibition by arylbenzofurans	Quantitative inhibitory data reported for related compounds	Supports exploration of neuroactive potential of arylbenzofuran scaffold	MFA not specifically tested
Vesely et al., 2021 [29]	Selected 2-arylbenzofurans	Level II/III: Related scaffold evidence	In vitro metabolism study	Colon in vitro model system	Reported metabolism of selected 2-arylbenzofurans in colonic conditions	Metabolic profiles reported; not MFA-specific	Useful for discussing possible metabolic fate of benzofuran-like compounds	Cannot be used as direct MFA pharmacokinetic evidence
Zhang et al., 2022 [30]	Mulberroside A	Level III: Contextual Morus constituent evidence	In vivo and in vitro metabolite identification	Rat metabolism study	Characterized mulberroside A metabolites using UHPLC-MS/network pharmacology	Analytical metabolism data reported	Useful only as contextual comparison in mulberry pharmacokinetic discussion	Different compound class; not MFA-specific
Kim et al., 2022 [15]	Mulberrofuran G	Level II: Related analogue evidence	Experimental antiviral study	SARS-CoV-2 spike RBD–ACE2 interaction assay	Reported blockade of spike RBD–ACE2 interaction by mulberrofuran G	Experimental activity data reported for analogue	Indirectly supports interest in antiviral activity within mulberrofuran family	Not MFA; activity cannot be assumed for MFA
Mei et al., 2012 [16]	Mulberroside A and oxyresveratrol	Level III: Contextual Morus constituent evidence	In vitro pharmacokinetic characterization	Absorption/metabolism-related assays	Reported pharmacokinetic features of mulberroside A and its bacterial metabolite	Quantitative PK-related data reported	Contextual comparator for discussing the lack of MFA PK data	Different scaffold and metabolism profile from MFA

Note: Level I indicates direct MFA-specific evidence, including studies in which purified Mulberrofuran A itself was isolated or tested. Level II indicates indirect evidence from structurally related mulberrofuran analogues or other arylbenzofurans. Level III indicates contextual evidence from Morus extracts, multi-component preparations, or broader Morus/benzofuran literature. Reported findings from Level II and Level III studies were used cautiously for comparative interpretation and hypothesis generation, not as definitive proof of MFA-specific pharmacological activity.

**Table 3:** Reported MIC Values and Antibacterial Spectrum.

Compound	Pathogen	Strain Type	MIC (μg/mL)	Reference
Mulberrofuran A	*Mycobacterium phlei*	Gram-positive	1.56	[3]
Mulberrofuran A	*Streptococcus faecalis*	Gram-positive	3.12	[3]
Mulberrofuran A	*Bacillus subtilis*	Gram-positive	3.12	[3]
Mulberrofuran A	*Staphylococcus aureus*	Gram-positive	6.25	[3]
Mulberry extract	*Escherichia coli*	Gram-negative	>100	[3]
Mulberrofuran G	*S. aureus* & *Enterococci*	Gram-positive	1.56–6.25	[3]
Kuwanon C	*S. aureus*	Gram-positive	5–30	[1]

Note: MIC values should be interpreted with caution, as antibacterial activity may vary depending on bacterial strain, inoculum size, assay conditions, endpoint definition, and compound purity.

**Table 4:** Preliminary structure–activity trends among Mulberrofuran A and Related Morus Constituents.

Type	Compound	Targets/Special Activity	Mechanism or Characteristics	Structure-Activity Relationship
Antibacterial Activity Comparison	Mulberrofuran A	*Mycobacterium phlei*	MIC: 1.56 μg/mL	Monomer + geranyl side chain = optimal membrane penetration
	Mulberrofuran G	*S. aureus* & *Enterococci*	MIC: 1.56–6.25 μg/mL	Diels-Alder adduct; polycyclic system enhances membrane interaction
	Kuwanon C	*S. aureus*	MIC: 5–30 μg/mL	Isoprenylated flavonoid; broader MIC range
Anti-inflammatory Mechanism Comparison	Mulberrofuran A	COX inhibition (HHT/TXB_2_ ↓) + 12-LOX indirect enhancement (12-HETE ↑)	Substrate shunting in AA metabolism	Monomeric structure; selective COX inhibition
	Mulberrofuran K	COX enzyme inhibition + NF-κB/ERK pathway transcriptional suppression	Double blockade strategy	Congener (inferred similar to MFA with possible additional phenolic OH)
	Mulberrofuran G	Endothelium-dependent vasodilation (likely NO production)	Antihypertensive (depressor effect)	Diels-Alder adduct; complex polycyclic system
Metabolic Disease (Diabetes/Dyslipidemia) Activity Comparison	Mulberrofuran A	α-glucosidase + PTP1B	Dual-target antidiabetic: delays glucose absorption + enhances insulin sensitivity	2-arylbenzofuran core + geranyl side chain enables dual inhibition; mimics PTP1B substrate conformation
	Mulberrofuran G	LDL oxidation inhibition + anti-atherogenesis	Antihyperlipidemic; prevents oxidative LDL modification	Diels-Alder adduct; multiple antioxidant sites from complex structure
	Albanol B	PTP1B inhibition (related congener)	Enhances insulin sensitivity (inferred from structural similarity)	Related to MFA; confirms PTP1B inhibition as class effect
	Mulberroside A	Requires colonic bacterial hydrolysis to aglycone	Prodrug form; slow-release, prolonged activity	Glycosylation blocks direct absorption; must be hydrolyzed to active aglycone
Antitumor Activity and Mechanism Comparison	Mulberrofuran A	CYP19A1 (estrogen synthesis), JAK2/STAT3 (proliferation/migration), GSK-3β/Wnt pathway	Aromatase inhibition + JAK2/STAT3 inhibition + GSK-3β inhibition	Monomer; high docking affinity to aromatase (binding energy −115.5 vs. lapatinib −96.9)
	Mulberrofuran G	HL-60 leukemia cells; mitochondrial pathway	Apoptosis induction	Diels-Alder adduct; polycyclic system
	Albanol B	Mitochondrial ROS production; in vivo tumor growth suppression	Anti-lung cancer	Dimeric structure related to MFG
Antioxidant Activity Comparison	Mulberrofuran B	Extremely high	Superior to MFA; significantly higher than Morusin	Number and position of phenolic hydroxyls optimized
	Mulberrofuran C	Extremely strong	~3× Trolox (reference standard)	Specific hydroxylation pattern enhances radical scavenging
	Mulberrofuran A	Moderate	Active but not the strongest among family	Basic 2-arylbenzofuran scaffold provides baseline antioxidant capacity
	Morusin	Weaker	Lower than Mulberrofuran B	Comparison baseline compound
Other Bioactivities and Specialized Functions	Mulberrofuran G	Anti-hepatitis B virus (HBV)	Inhibits viral replication	Diels-Alder adduct; complex structure enables viral protein interaction
	Mulberrofuran G	Anti-SARS-CoV-2	Blocks Spike protein S1-ACE2 interaction	Polycyclic system provides multiple protein binding sites
	Mulberrofuran C	Anti-HAV 3C protease	In silico predicted inhibition	Monomeric structure; distinct from MFG’s antiviral mechanism
	Mulberrofuran F	Hypotensive (depressor)	Intravenous injection in rabbits: 26 mmHg reduction at 0.1 mg/kg	Diels-Alder type adduct; related to MFG

Note: The trends summarized in this table represent preliminary comparative observations and should not be interpreted as definitive SAR rules, because the available evidence derives from different compounds, assay systems, and levels of validation.

## Data Availability

No new data were created or analyzed in this study. Data sharing is not applicable to this article.

## Supplementary Materials

The following supporting information can be downloaded at: https://www.mdpi.com/article/10.3390/molecules31101755/s1, Table S1: Methodological Quality Assessment; Table S2: In Silico vs. Experimental Evidence Comparison. References [3,6,8,11,14,22,23,38,41] are cited in the Supplementary Materials.

## Abbreviations

The following abbreviations are used in this manuscript:

12-HETE	12-hydroxyeicosatetraenoic acid
12-HHT	12-hydroxy-5,8,10-heptadecatrienoic acid
12-HPETE	12-hydroperoxyeicosatetraenoic acid
12-LOX	12-lipoxygenase
AA	arachidonic acid
ABTS	2,2′-azino-bis(3-ethylbenzothiazoline-6-sulfonic acid)
ACE2	Angiotensin-converting enzyme 2
ADME	Absorption, Distribution, Metabolism, Excretion
ATF4	Activating transcription factor 4
AUC	Area under the curve
CHOP	C/EBP homologous protein
Cmax	Maximum plasma concentration
COX	Cyclooxygenase
DPPH	2,2-diphenyl-1-picrylhydrazyl
EC50	Half maximal effective concentration
EGFR	Epidermal growth factor receptor
eIF2α	Eukaryotic translation initiation factor 2 alpha
ER (cancer)	Estrogen receptor
ER (cell)	Endoplasmic reticulum
ERK1/2	Extracellular signal-regulated kinases 1/2
EV-A71	Enterovirus A71
GLUT4	Glucose transporter type 4
HER2	Human epidermal growth factor receptor 2
HHT	12-hydroxy-5,8,10-heptadecatrienoic acid
HMBC	heteronuclear multiple bond correlation
HPLC	High-performance liquid chromatography
IC50	Half maximal inhibitory concentration
IL-1β	Interleukin-1 beta
IL-6	Interleukin 6
iNOS	Inducible nitric oxide synthase
IRE1α	Inositol-requiring enzyme 1 alpha
JAK2	Janus kinase 2
LDL	Low-density lipoprotein
LOX	lipoxygenase
LPS	Lipopolysaccharide
MAPK	Mitogen-activated protein kinase
MFA	Mulberrofuran A
MIC	Minimum Inhibitory Concentration
MMP-2	Matrix metalloproteinase-2
NF-κB	nuclear factor kappa-B
NOESY	nuclear overhauser effect spectroscopy
NSAIDs	non-steroidal anti-inflammatory drugs
PERK	Protein kinase R-like endoplasmic reticulum kinase
PGG2	Prostaglandin G2
PGH2	Prostaglandin H2
PK	Pharmacokinetics
PLA2	Phospholipase A2
PTP1B	Protein Tyrosine Phosphatase 1B
ROS	Reactive oxygen species
SAR	structure-activity relationship
SARS-CoV-2	Severe acute respiratory syndrome coronavirus 2
STAT3	Signal transducer and activator of transcription 3
SULT	Sulfotransferase
T2DM	Type 2 diabetes mellitus
TEAC	Trolox equivalent antioxidant capacity
Tmax	Time to maximum plasma concentration
TNF-α	Tumor necrosis factor alpha
TXB2	thromboxane B2
UGT	UDP-glucuronosyltransferase
UV	Ultraviolet
XBP1	X-box binding protein 1
