# Supplementary material for: Mulberrofuran A: A Multifunctional 2-Arylbenzofuran Flavonoid—Insights into Pharmacological Actions, Molecular Mechanisms, and Therapeutic Potential
Source: Molecules. 2026 May 20;31(10):1755. doi: 10.3390/molecules31101755 (PMC13210023; doi:10.3390/molecules31101755)
Supplement: Supplementary file 1 [file molecules-31-01755-s001.zip › molecules-4255402-supplementary.pdf]

Table S1: Methodological Quality Assessment

Reference	Study Type	Statistical Reporting	Sample Size	Reproducibility	Risk of Bias	Evidence Level
Nomura 1978[6]	Phytochemical isolation	None (structural study)	N/A	Low	Low	Level I
Sohn 2004[3]	Antimicrobial MIC	MIC reported; no replicate statistics	Not reported	Moderate	Unknown	Level I
Kimura 1986[41]	Rat platelet AA metabolism	Qualitative; no exact P-values	n=3–5 per group	Low-Moderate	Unknown	Level I
Sureshan 2024[14]	Molecular docking	Docking scores reported	N/A	Low-Moderate	Protocol not validated	Level IV
Geng 2012 (MFG)[23]	Enzyme assay + cellular	IC50 ± SD; MTT/Western blot statistics	Not clearly reported	High	Low	Level II

Note: Level I indicates direct MFA-specific evidence, including studies in which purified Mulberrofuran A itself was isolated or tested. Level II indicates indirect evidence from structurally related mulberrofuran analogues or other arylbenzofurans. Level III indicates contextual evidence from Morus extracts, multi-component preparations, or broader Morus/benzofuran literature. Level IV indicates computational/in silico evidence.

Table S2: In Silico vs. Experimental Evidence Comparison

Target	In Silico Evidence (MFA)	Experimental Evidence (MFA or Analogue)	Gap Assessment
PTP1B	Docking score [8]	Mulberrofuran G: IC50 = 0.57 ± 0.04 μM; cellular glucose uptake validated [8]	MFA not tested
α-glucosidase	Indirect from stilbenoids	Mulberrofuran G: mixed-type inhibition confirmed [8]	MFA not tested
3C Protease	Docking score reported [14]	None	No biochemical assay
Aromatase (CYP19A1)	Docking predicted [22,38]	None	No enzyme assay
GSK-3β	Docking predicted [11]	Arylbenzofuran scaffold inhibition confirmed; MFA not tested	MFA not tested
COX/LOX	AA metabolite modulation in rat platelets [3]	Rat platelet homogenate — ex vivo assay only	No in vivo confirmation
Antibacterial	MIC reported[3]	MIC in 4 bacterial strains [3]	No time-kill kinetics, no in vivo infection model
